# Supplementary material for: Daily humidity oscillation regulates the circadian clock to influence plant physiology
Source: Nat Commun. 2018 Oct 16;9:4290. doi: 10.1038/s41467-018-06692-2 (PMC6191426; doi:10.1038/s41467-018-06692-2)
Supplement: Supplementary file 1 — Supplementary Information [file 41467_2018_6692_MOESM1_ESM.pdf]

Supplementary Information for

Daily humidity oscillation regulates the circadian clock to influence plant physiology

Musoki Mwimba, Sargis Karapetyan, Lijing Liu, Jorge Marqués, Erin M. McGinnis, Nicolas E. Buchler & Xinnian Dong*

*Corresponding author. E-mail: xdong@duke.edu

Supplementary Figure 1

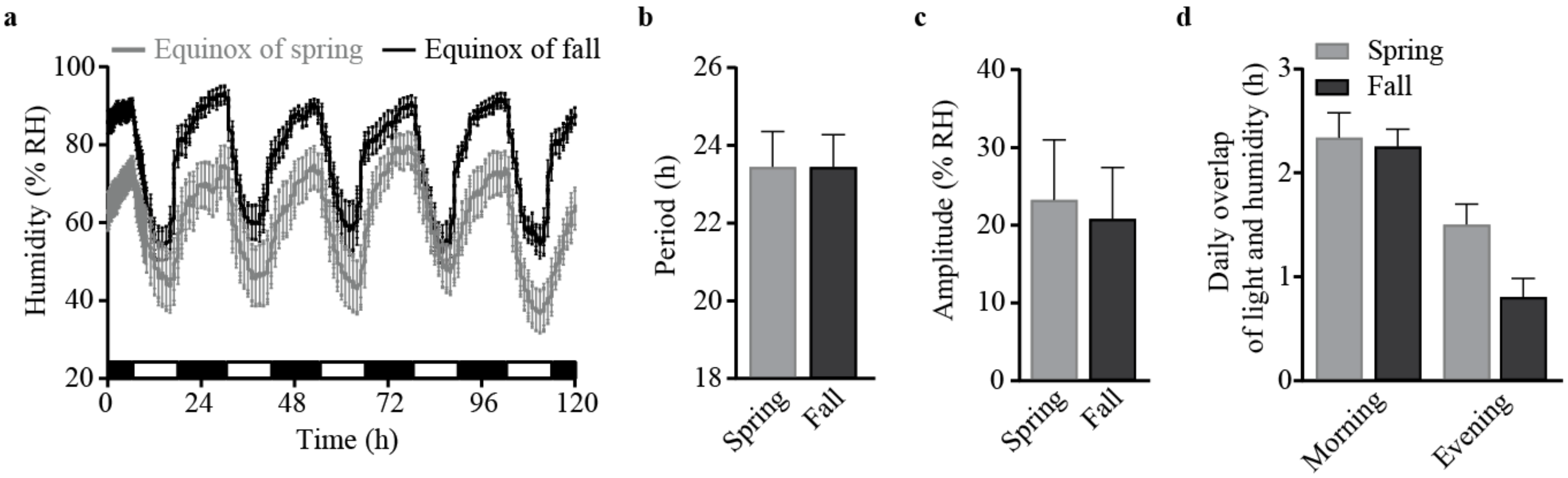

Supplementary Figure 1 | Quantitative characterization of humidity oscillation at Harvard Forest. (**a**) Rhythms of humidity during spring equinox (grey; March 19-23, 2005-2016) and fall equinox (black; September 21-24, 2005-2016) at Harvard Forest. Black bars represent nights; White bars, days; Data are shown as mean ± s.e.m.; n=12 years. (**b-d**) Calculated periods (b), amplitudes (half of the range) (c) and the daily overlaps of high humidity and light in the morning and low humidity and dark in the evening (d). Data are shown as mean ± s.d.; n=12 years

Supplementary Figure 2

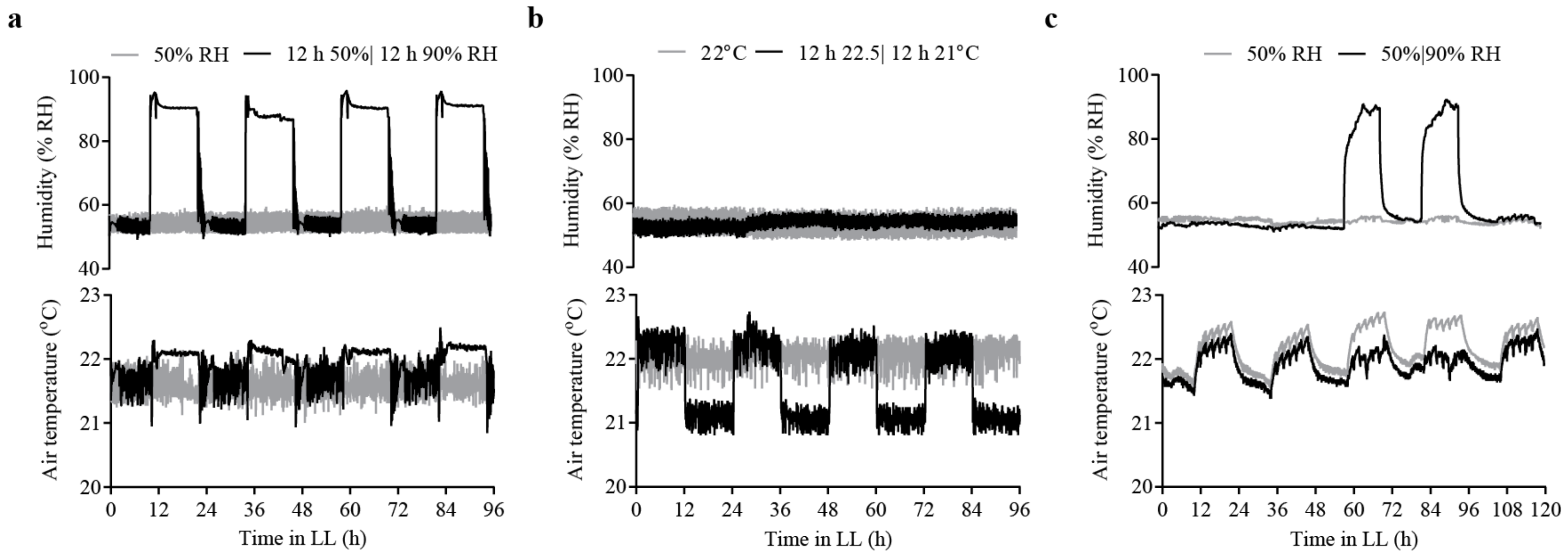

Supplementary Figure 2 | Control of humidity in the Percival and the imaging chambers. (**a** and **b**) Traces of humidity (top) and air temperature (bottom) in two identical Percival growth chambers with either oscillating humidity (a) or oscillating temperature (b). In (a), humidity was set to oscillate between 50% RH and 90% RH in one chamber (black) and kept constant at around 50% RH in the other chamber (grey) while temperature was kept at 22°C. In (b), air temperature was set to oscillate between 22.5°C and 21°C in one chamber (black) and kept constant at 22°C in the other chamber (grey) while humidity was set at 50% RH. (**c**) Traces of humidity (top) and air temperature (bottom) in the two compartments of the custom-built imaging chamber. Humidity was set to oscillate between 50% RH and 90% RH in one compartment (black) and kept constant at around 50% RH in the other compartment (grey) while temperature was maintained at around 22°C.

Supplementary Figure 3

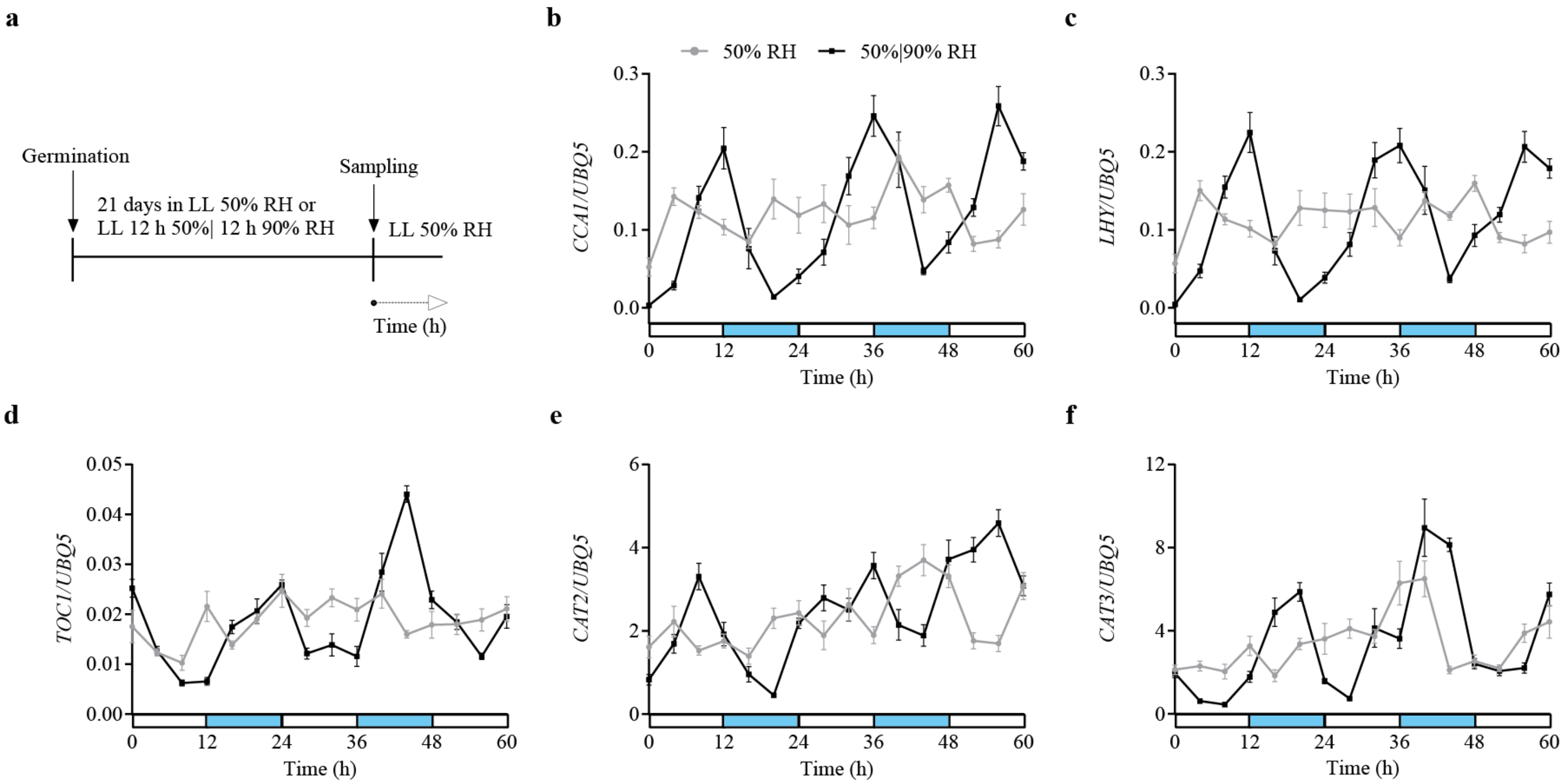

Supplementary Figure 3 | Humidity oscillation entrains the clock in LL. (**a**) Workflow describing the entrainment experiment in (b-f). (**b-f**) mRNA rhythms in three-week-old WT plants which were grown in LL 50 % RH (grey) or LL 12 h 50% RH | 12 h 90% RH (black) and then released into free-running conditions at time 0 h. mRNA levels were analyzed by qPCR and normalized against *UBIQUITIN 5* (*UBQ5*). White bars, 50% RH; light blue bars, subjective 90% RH. Data are shown as mean ± s.d. of five biological replicates.

Supplementary Figure 4

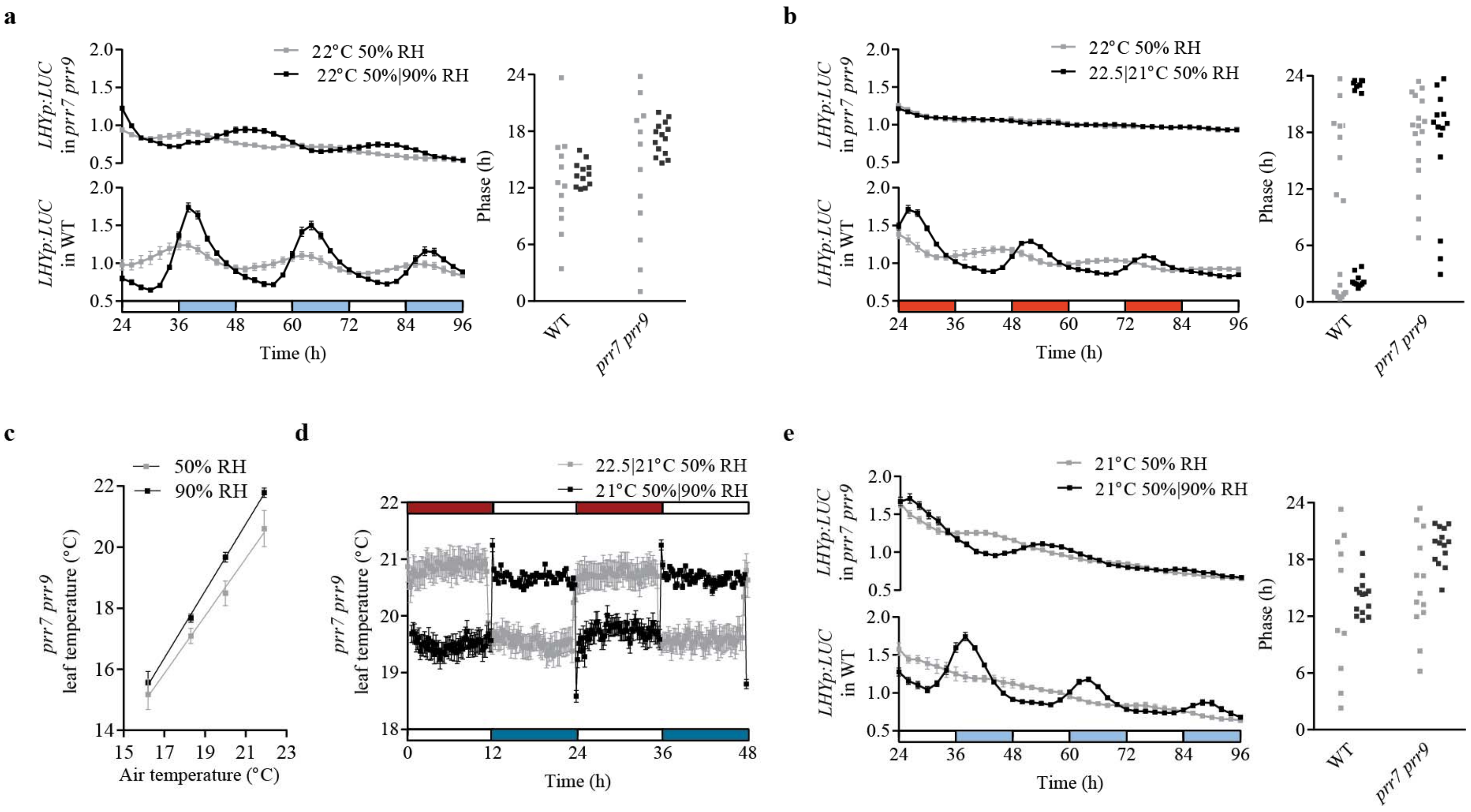

Supplementary Figure 4 | Humidity, but not temperature, entrains the *prr7 prr9* circadian clock in LL. (**a**) Luciferase reporter *LHYp:LUC* activity rhythms in 3-week-old *prr7 prr9* mutant (top) or WT (bottom) and their calculated phases (right). Plants were grown under LL 22°C with either constant 50% RH (grey) or 12 h 50% RH| 12 h 90% RH (black). White bars represent 50% RH, light blue bars, subjective 90% RH; Plants were moved to free running conditions at time 0 h. Data are shown as mean ± s.e.m.; $n \geq 20$. Relative amplitude error values of ≤ 0.7 were considered rhythmic. The experiment was repeated two times with similar results. (**b**) Luciferase reporter *LHYp:LUC* activity rhythms in 3-week-old *prr7 prr9* mutant (top) or WT (bottom) and their calculated phases (right). Plants were grown under LL at 50% RH with either constant 22°C (grey) or 12 h 22.5°C| 12 h 21°C (black). White bars represent 21°C, light red bars, subjective 22.5°C; Plants were moved to free-running conditions at time 0 h. Data are shown as mean ± s.e.m.; $n \geq 20$. Relative amplitude error values of ≤ 0.7 were considered rhythmic. The experiment was repeated three times with similar results. (**c**) *prr7 prr9* leaf temperature and air temperature relationship at 50% RH (grey) and at 90% RH (black). Data are shown as mean ± s.d.; $n \geq 288$. Black and grey lines show the best linear fit. (**d**) *prr7 prr9* leaf temperature measurements in response to 12 h 22.5°C| 12 h 21°C (grey) and 12 h 50% RH| 12 h 90% RH (black). Top red bars, 22.5°C; top white bars, 21°C; bottom blue bars, 90% RH, bottom white bars, 50% RH. (**e**) Luciferase reporter *LHYp:LUC* activity rhythms in 3-week-old *prr7 prr9* mutant (top) or WT (bottom) and their calculated phases (right). Plants were grown under LL 21°C with either constant 50% RH (grey) or 12 h 50% RH| 12 h 90% RH (black).

White bars represent 50% RH, light blue bars, subjective 90% RH; Plants were moved to free-running conditions at time 0 h. Data are shown as mean ± s.e.m.; n ≥ 20. Relative amplitude error values of ≤ 0.7 were considered rhythmic. The experiment was repeated three times with similar results.

Supplementary Figure 5

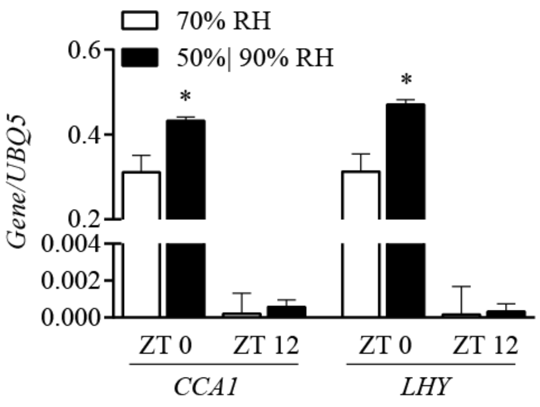

Supplementary Figure 5 | Simulated natural conditions increase the amplitudes of *CCA1* and *LHY*. *CCA1* and *LHY* mRNA levels in 3-week-old WT plants grown under simulated natural conditions (black) or in free-running conditions (white). Plant samples were collected at dawn (ZT 0 h) or dusk (ZT 12 h) from which mRNA was extracted, analyzed by qPCR and normalized against *UBIQUITIN 5* (*UBQ5*). Data are shown as mean ± s.d. of four experimental replicates combined using linear mixed effect model (lme4) with experiment as random effects; *t*-test; *$p < 0.05$.

Supplementary Figure 6

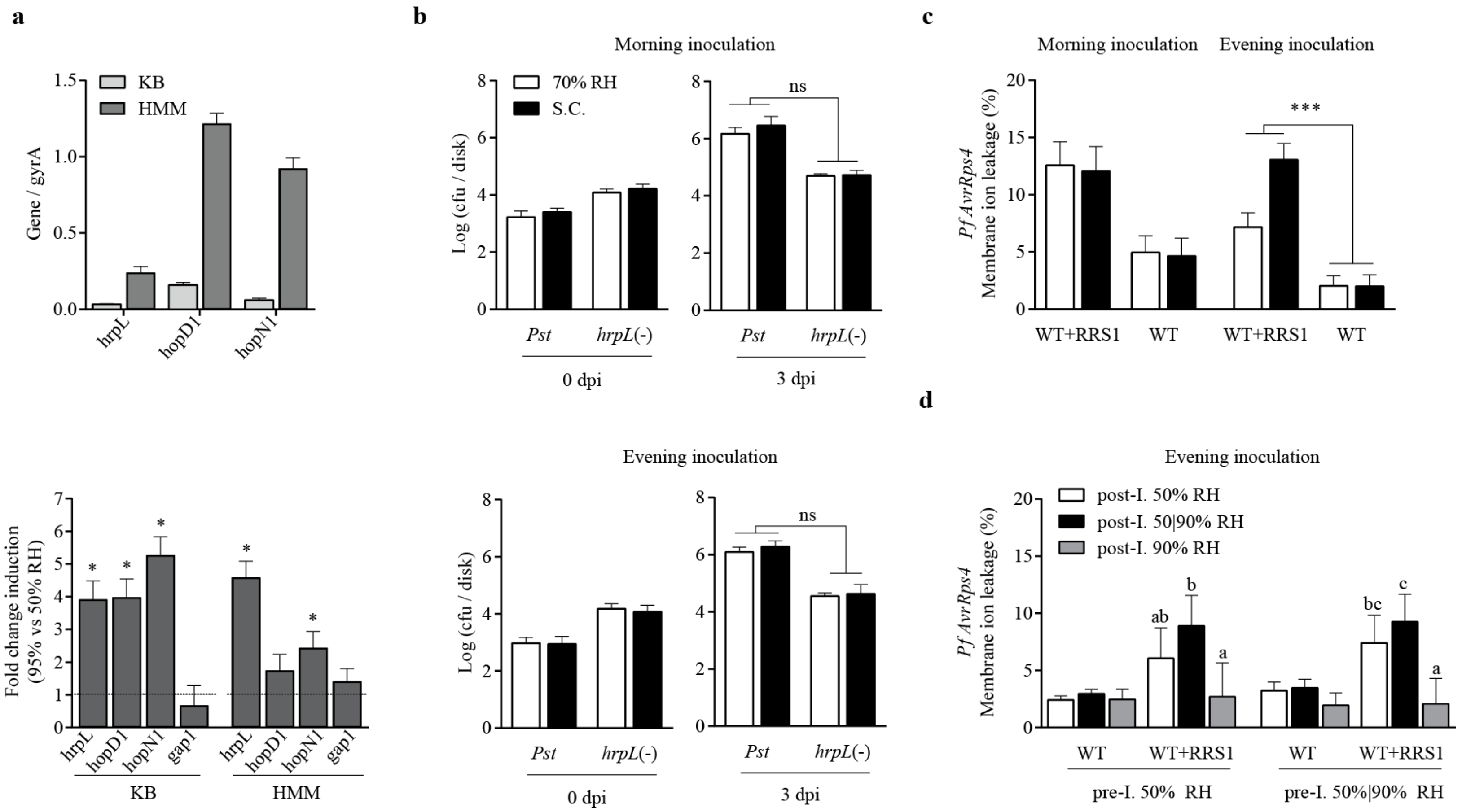

Supplementary Figure 6 | Influence of humidity oscillation on plant-microbe interactions. (**a**) Expression of T3SS genes (top) and their fold induction after 6 h in 95% RH (bottom) in bacteria grown on plates. Gene expression relative to DNA gyrase A (gyrA) was determined before fold change was calculated. KB, King's B medium plates; HMM, hrp minimal medium plates. Data are shown as mean ± s.d. of five experimental replicates combined using linear mixed effect model (lme4) with experiment as random effects; *t*-test performed on relative gene expression values; *p < 0.05. (**b**) Quantification of bacterial growth in 3-week-old plants after inoculation with *Pst* (OD_{600nm}= 0.001) or *Pst hrpL(-)* (OD_{600nm}= 0.01) in the morning (ZT 1 h) (top) or in the evening (ZT 13 h) (bottom). Plants were grown under LD with constant 70% RH (white) or simulated natural conditions (black). cfu, colony-forming unit; dpi, days post inoculation. Data are shown as mean ± s.d.; n ≥ 7; two-way ANOVA; ns, not significant. This experiment was repeated twice with similar results. S.C., simulated natural conditions. (**c**) Ion leakage in 3-week-old WT and WT carrying the RPS4 immune receptor, RRS1. Plants were grown under LD with 70% RH (white) or simulated natural conditions (black) and infiltrated in the morning (ZT 1 h) (left) or in the evening (right) with *Pf AvrRps4* (OD_{600nm} = 0.02). Ion leakage measurement was performed at 36 h post inoculated when ETI-associated cell death was expected to occur. Data are shown as mean ± s.d. of three experimental replicates representing at least 18 plants. Data were combined using linear mixed effect model (lme4) with experiment as random effects; two-way ANOVA; ***p < 0.001. (**d**) Ion leakage measurement performed at 36 h post *Pst AvrRps4* inoculation of 3-week-old plants grown under LD with 70% RH or simulated natural conditions. Leaves were infiltrated with bacteria at OD_{600nm} = 0.02 in the evening (ZT 13 h). Data are shown as mean ±

s.d. of three experimental replicates representing at least 18 plants from a single parent line. Data were combined using linear mixed effect model (lme4) with experiment as random effects; *t*-test, the same letters above bars denote values that were statistically similar.

Supplementary Tables

Supplementary Table 1 | Pearson correlation between humidity and temperature

**Growth chamber**		
	Temperature	Soil moisture
50% RH	0.03146352	NA
50%|90% RH	0.007503736	NA
**Imaging Chamber**		
	Temperature	Soil moisture
50% RH	-0.006342905	0.3479596
50%|90% RH	0.2327999	-0.1281912
**Harvard Forest**		
	Temperature	Soil moisture
50%|90% RH	-0.90145	NA

Supplementary Table 2 | Periodicity analysis of luciferase activity rhythms

Figure	Reporter	Back-ground	Conditions	Total Number	FFTNLS analysis			LS analysis		
					Rhythmic Traces	Rhythmic Fraction	Sync. Index	Rhythmic Traces	Rhythmic Fraction	Sync. Index
Fig. 2a-d	CCA1p:LUC	WT	50% RH	20	8	8/20=0.40	0.13	7	7/20=0.40	0.15
		WT	50%|90% RH	21	16	16/21=0.76	0.56	11	11/21=0.55	0.40
	LHYp:LUC	WT	50% RH	20	20	20/20=1.00	0.13	20	20/20=1.00	0.25
		WT	50%|90% RH	20	20	20/20=1.00	1.00	20	20/20=1.00	0.97
	TOC1p:LUC	WT	50% RH	20	20	20/20=1.00	0.28	20	20/20=1.00	0.24
		WT	50%|90% RH	20	20	20/20=1.00	0.94	20	20/20=1.00	0.99
	CAT2p:LUC	WT	50% RH	17	15	15/17=0.88	0.17	13	13/17=0.76	0.15
		WT	50%|90% RH	17	17	17/17=1.00	0.95	17	17/17=1.00	0.96
	CAT3p:LUC	WT	50% RH	18	16	16/18=0.89	0.25	15	15/18=0.83	0.04
		WT	50%|90% RH	18	15	15/18=0.83	0.83	14	14/18=0.77	0.75
	CCR2p:LUC	WT	50% RH	17	13	13/17=0.76	0.33	10	10/17=0.58	0.21
		WT	50%|90% RH	17	11	11/17=0.64	0.64	9	9/17=0.53	0.47
Suppl. Fig. 4a	LHYp:LUC	WT	50% RH	12	12	12/12=1.00	0.45	12	12/12=1.00	0.43
		WT	50%|90% RH	12	12	12/12=1.00	0.95	12	12/12=1.00	0.99
		prr7 prr9	50% RH	15	13	13/15=0.86	0.15	9	9/15=0.60	0.06
		prr7 prr9	50%|90% RH	15	14	14/15=0.93	0.85	15	15/15=1.00	0.98
Suppl. Fig. 4b	LHYp:LUC	WT	22°C	18	17	17/18=0.94	0.47	18	18/18=1.00	0.45
		WT	22.5|21°C	20	18	18/20=0.90	0.81	18	18/20=0.90	0.86
		prr7 prr9	22°C	20	17	17/20=0.85	0.42	10	10/20=0.50	0.38
		prr7 prr9	22.5|21°C	18	15	15/18=0.83	0.37	6	6/18=0.33	0.21
Suppl. Fig. 4e	LHYp:LUC	WT	50% RH	12	10	10/12=0.83	0.12	8	8/12=0.75	0.25
		WT	50%|90% RH	16	16	16/16=1.00	0.79	15	15/16=0.93	0.85
		prr7 prr9	50% RH	20	16	16/20=0.80	0.23	7	7/20=0.35	0.13
		prr7 prr9	50%|90% RH	20	15	15/20=0.75	0.56	17	17/20=0.85	0.62

Supplementary Table 3 | qPCR primer pairs

Genes	Forward primer	Reverse primer
CCA1	TGACCGGTCCTCGTGTGGCT	ACTGCGGCGTGCATTGGACT
LHY	CGCTGCTTCGGTCTGGCCTT	TGTAGCAGCGGCAATGGCAGT
TOC1	AATAGTAATCCAGCGCAATTTCTTC	CTTCAATCTACTTTTCTTCGGTGCT
UBQ5	GACGCTTCATCTCGTCC	GTAAACGTAGGTGAGTCC
HrpL	GCCGATCAGATTCAGATGCTCAG	TTACGCAGGGCTTCAAGAAACAC
HopD1	GCCCAGCAATCCCATCCTAAAAG	GCGTTGGTGTTCGGCATTAGC
HopN1	CCATTGGAGGGGCATGAAGAGG	GTTGTACCTGCTCGGGACTGG
GyrA	AGGCAAGTATTTCCTGTCGCC	CTGGTACTCACCCAGCAGTTTTT
Gap1	CCGCAAGGTGATTATCTCAGC	TGGAGATGATCTGGTGCGACT
