# Supplementary material for: Daily humidity oscillation regulates the circadian clock to influence plant physiology
Source: Nat Commun. 2018 Oct 16;9:4290. doi: 10.1038/s41467-018-06692-2 (PMC6191426; doi:10.1038/s41467-018-06692-2)
Supplement: Supplementary file 3 — Description of Additional Supplementary Files [file 41467_2018_6692_MOESM3_ESM.pdf]

Description of Additional Files:

Supplementary Data 1: Excel file containing raw data for all figures in Mwimba et al.
